# On the achievable consistency of glycan distribution in biomanufacturing of therapeutic mAbs

**DOI:** 10.1038/s44334-025-00058-5

**Published:** 2026-01-06

**Authors:** Hongbin Zhu, Joshua Shipman, Weiming Ouyang, Kang Chen

**Affiliations:** 1 Division of Pharmaceutical Quality and Research II, Office of Pharmaceutical Quality Research, Office of Pharmaceutical Quality, Center for Drug Evaluation and Research, U.S. Food and Drug Administration, St. Louis, MO, USA.; 2 Division of Pharmaceutical Quality and Research III, Office of Pharmaceutical Quality Research, Office of Pharmaceutical Quality, Center for Drug Evaluation and Research, U.S. Food and Drug Administration, Silver Spring, MD, USA.; 3 Division of Pharmaceutical Quality and Research II, Office of Pharmaceutical Quality Research, Office of Pharmaceutical Quality, Center for Drug Evaluation and Research, U.S. Food and Drug Administration, Silver Spring, MD, USA.

## Abstract

Recombinant monoclonal antibody (mAb) therapeutics exhibit lot-to-lot glycosylation variation influenced by manufacturing processes, which remains underexplored publicly. This study analyzed five years of trastuzumab and adalimumab lots, revealing up to 4% range of variation in glycan relative abundance and significant change or drift of ~1% by ANOVA, levels unlikely to affect mAb efficacy or safety. The results suggest that modern manufacturing can maintain consistent glycan profiles within realistically achievable ranges.

Monoclonal antibody (mAb) drugs are large, multi-domain immunoglobulin G (IgG) proteins consisting of two light and two heavy chains that form one crystallizable (Fc) domain and two antigen-binding (Fab) domains. The Fc domain typically engages effector cells, while the Fab domains bind target antigens. During recombinant production, the Fc domain, and in more complex mAbs the Fab domain (*e.g*., cetuximab)^1^, undergo N-glycosylation as part of post-translational modifications (PTMs) in the endoplasmic reticulum (ER) and Golgi apparatus. This process involves the addition of glycans through the actions of various glycosyltransferases and glycosidases^2^. Initially, within the ER a high-mannose structure is added, which could be trimmed by glycosidases to the trimannosyl core structure composed of two N-acetylglucosamine (GlcNac) and three mannose (Man) units. Upon transfer to the Golgi complex, further processing occurs, where terminal mannose residues are removed and other sugars, such as GlcNAc, fucose (Fuc), galactose (Gal), and N-acetylneuraminic acid (Neu5Ac), are added. The final product is typically a complex glycan with GlcNAc at the 1–3 and 1–6 branches, which is the predominant form of glycan found in mAbs. Other glycan species are high-mannose (HM), afucosylated complex and hybrid type, which has the 1–3 branchof GlcNAc and the 1–6 branch of mannose (Fig.1). Glycan structures modulate mAb therapeutic efficacy, safety and serum life^3–7^; thus, consistent glycan distribution is critical for maintaining the therapeutic features of mAbs^8–10^. Product glycan composition depends on host cell type, culture components, growth conditions, and purification. Consequently, glycan distribution can vary intrinsically from batch to batch, and may also shift due to manufacturing drift or intentional process changes^11,12^. Whether modern biomanufacturing can maintain glycan distribution within a narrow range over years remains to be addressed, but publicly available analytical data to assess the consistency under current processes are limited^10^. In this study, changes in glycan distribution of trastuzumab and adalimumab were measured using commercial drug lots purchased between 2018 and 2022 (Table 1, S1, S2). The mAbs were characterized using a high-resolution glycan mapping protocol based on liquid chromatography - parallel reaction monitoring mass spectrometry (LC–PRM-MS) (Table S3)^13^. Glycan relative abundance changes across all the mAb drug lots were reported as ranges, likely reflecting inherent manufacturing variability^10^. To assess potential process drift, mAb lots were further grouped by expiry date (Table 1; two to three lots per group; six groups in total), and one-way Analysis of Variance (ANOVA) was performed to assess significant differences in glycan abundance changes^10^.

For trastuzumab, 39 glycans were identified and quantified and 17 of them showed significant changes among the six groups (ANOVA *p* < 0.05; Table S4). The three predominant glycans (>10%), complex type FA2, FA2[6]G1, and FA2[3]G1, together accounted for about 80% of the total glycan content, calculated using the most recent lot data (Table S5). FA2 and FA2[6]G1 had a changing range of 4.4% and 2.0%, respectively (Fig 2.), above the experimental precision of 0.3% or less (Table S5) but not statistically significant among the groups (*p* > 0.05) (Fig 3.). In contrast, FA2[3] G1 had a range of change of only 1.1%, but was significant (*p* < 0.05), indicating a drifting decrease in galactosylation on the 1–3 branch among the groups. The five major glycans (1–10%) including FA2G2, A2, M5, A2[6]G1 and FA1, constituted 16% of the total glycan content. Among them, FA2G2 had the largest range of change of 1.6%, but it was not significant; A2 and M5 were identified to be significantly increased. The increase ranges of 0.6% for A2 and 0.8% for M5, along with a 1.1% decrease in FA2[3]G1, suggest reduced glycan processing from HM or afucosylated/non-galactosylated structures to more complete complex glycan types among the product lots purchased over the five years. The next 13 minor glycans (0.1–1%) constituted 3.8% of total glycans and 6 of the 13 were significantly changed among the groups. Within the minor glycans, FA1G1 and A2[3]G1 had the largest range of change of 0.2%, but they were not significant. The significantly changed minor glycans were FA2[6]G1S1, FA3G1, FA1G1S1, FA2[3]G1S1, A1, A1G1 and FA2[3]G1S1. The decreases in the sialylated glycans, e.g., 0.1% in FA2[6]G1S1 and 0.08% in FA1G1S1, were possibly due to fewer substrates of galactosylated glycans or lower enzyme activity; and the increases in the hybrid glycan, e.g., 0.08% in A1 and 0.03% in A1G1, were consistent with the reduced processing observed in the predominant and major glycans. The complex glycan FA3G1 increased by 0.05%, suggesting enhanced galactosylation activity, in contrast to the decreasing trends of FA2[3]G1 and FA2[6]G1. These differences may reflect distinct enzymatic pathways for galactosylation of FA3 versus FA2 glycans, consistent with the recent findings^14^. The remaining 18 trace glycans, each below 0.1% abundance, together accounted for only 0.47% of the total glycan content. Although 8 of the 18 showed significant changes among groups, they were not further analyzed due to their low abundances.

For adalimumab, a total of 38 glycans were identified and quantified and 25 of them had significant changes among the six groups (Table S6). The two predominant glycans of FA2 and FA2[6]G1, constituting 82% of total glycan content calculated using the most recent lot data (Table S7), had ranges of change of 1.9% and 1.2%, respectively (Fig 2.). The ranges of change were above experimental precision of 0.2% or less (Table S7), but only the change of FA2[6]G1 was deemed statistically significant per ANOVA (*p* < 0.05) (Fig 4.). The five major glycans (1–10%) of FA2[3]G1, M5, FA1, FA2G2 and FA1G1 constituted 14% of the total glycan content, and FA2[3]G1, M5 and FA1 had the largest range of change of 0.5%. Among the major glycans, FA2[3]G1, FA1 and FA2G2 were significantly changed. BothFA2[6]G1andFA2[3]G1 showeda concave upwardtrend fromGroup I to Group VI, mirroring the convex curve of FA2. This indicated that galactosylation drove the redistribution among the three most abundant glycans, i.e., when FA2 reached its minimum in Group III, FA2[3]G1 and FA2[6]G1 peaked. Similarly, the change patterns of FA1 and FA2G2 followed those of FA2 and FA2[6]G1/FA2[3]G1, respectively, confirming variation in galactosylation activity among the predominant glycans. The next 13 low abundance minor glycans (0.1–1%) constituted 3.6% of the total glycan content and 10 of the 13 exhibited significant changes. Overall, the decreases of 0.1% in M5, 0.1% in A2, 0.05% in M3, 0.3% in FM3 between Group I and VI, along with the decreasing trend of M6(2) and M7(3), balanced the Group-I to VI increase of complex glycans of 0.4% in FA2[6] G1 and 0.1% in FA2[3]G1, demonstrating increased processing from trimannosyl core glycans M3 or FM3, HM and afucosylated complex A2 to major complex glycans (Fig 1.). The ranges of changes in other minor glycans, FA2B, FA3, A2[6]G1, FA3G1, and FM5A1, were below 0.1%; therefore, they were not discussed. The remaining 18 trace glycans were less than 0.1% in abundance and constituted only 0.8% of the total glycan content. Although 11 of the 18 showed significant changes among groups, they were not further analyzed due to their low abundances.

In summary, the predominant glycan FA2 exhibited the greatest lot-tolot fluctuation, ranging from 34 to 39% (4.4% in range) in trastuzumab and 65–67% (1.9% in range) in adalimumab over the five-year period. Moreover, ANOVA revealed statistically significant changes in FA2[3]G1 for trastuzumab (11–12%, 1.1% in range) and FA2[6]G1 for adalimumab (16–17%, 1.2% in range), indicating a drift in these glycans across the lots purchased during this time frame. In trastuzumab glycan profile, decreases in galactosylated and sialylated glycans, alongside increases in high mannose (HM) and hybrid glycans, suggest reduced processing toward complex forms. Conversely, adalimumab showed increased processing from HM and hybrid to complex glycans, with elevatedgalactosylationand fucosylation within complex glycans as well. Overall, the glycan distribution changes followed the established bio-synthetic pathway (Fig 1.). However, the exact causes remain unknown due to the lack of publicly available manufacturing information for the analyzed lots.

In conclusion, among nearly 40 quantified glycans in commercial mAb products, maximum fluctuations of ~4% in FA2 and drifts of ~1% in FA2[3] G1 and FA2[6]G1 were observed, underscoring the high level of glycan profile consistency attainable with modern biomanufacturing and analytical methods over a five-year period^15,16^. These variations are modest compared to the previously reported abrupt shifts, suchas a 3-fold increase in G0 (0.4% to 1.2%) for rituximab and a 20% drop in G2F (50% to 30%) for etanercept, likely attributable to manufacturing changes^10^. In the absence of deliberate process modifications, the observed range of variation (<4%) may serve as a realistic benchmark for evaluating future process changes or biosimilar interchangeability^17–20^. Notably, the safe and effective use of these product batchesduring the study period suggests that, although some glycan changes were statistically significant, they are unlikely to meaningfully affect therapeutic outcomes^20,21^.

## Methods

### mAb drug products and chemicals

Ammonium formate solution and GlycoWorks RapiFluor-MS N-Glycan kits (Part# 176003606) were obtained from Waters. The mAb drug products of trastuzumab and adalimumab were sourced from the U.S. market (Tables S1 and S2). All mAb drug samples were diluted in water to a concentration of 2 mg/mL. The N-glycan release, labeling, and cleanup were performed in duplicate for each drug lot (Tables S1 and S2) following the manufacturer’s protocol^22^. Digestion buffer was made by dissolving one vial of RapiGest SF (10 mg) in 0.2 mL of GlycoWorks Rapid Buffer to form RapiGest buffer. A 7.5 μL of diluted mAb solution (2 mg/mL) was aliquoted and mixed with 15.3 μL of water and 6 μL of RapiGest buffer. The mixture was incubated at 90 °C in a heating block for three minutes. A 1.2 μL aliquot of Rapid PNGase F was added to the mAb sample to release N-glycan, which was incubated at 50 °C for five minutes then cooled for three minutes. Fluorescent labeling of released glycans was performed using GlycoWorks RapiFluor-MS labeling reagent, which was prepared by dissolving 23 mg of GlycoWorks RapiFluor-MS Reagent Powder in 0.335 mL of anhydrous dimethylformamide (DMF). Briefly, 12 μL of the labeling reagent was added to each PNGase F digested mAb sample and incubated at room temperature (20–25 °C) for 5 min. Each sample was then diluted with 0.358 mL of acetonitrile (ACN). The solutions of fluorescence labeled N-glycan molecules were purified using a GlycoWorks μElution plate. Wells were conditioned with 0.2 mL 18 mΩ water, then equilibrated with 0.2 mL of 85:15 (v/v) ACN:water. The diluted labeled glycan samples were loaded onto the treated wells, then wells were washed with two 0.6 mL of 90:9:1 (v/v/v) ACN:water:formic acid. Glycans were eluted with three 30 μL volumes of solid phase extraction (SPE) Elution Buffer composed of 95:5 (v/v) 200 mM ammonium acetate:ACN. The eluted samples were dried down and stored at −80 °C. Immediately prior to analysis samples were resuspended in 50 μL solution composed of 25:75 (v/v) water: Glycoworks sample diluent, which was composed of 32:68 (v/v) DMF:ACN. For precision test, six preparations were repeated to derive standard deviation (Table S5 and S7), and the precision results were not included for ANOVA analysis.

### LC-MS

A published LC-MS protocol based on parallel reaction monitoring (PRM) was adopted^13^. Experiments were performed on Waters Acquity BEH Amide column installed on Vanquish Horizon Binary UHPLC coupled to an Orbitrap Exploris 480 with a heated electrospray ionization (HESI) probe using positive ionization (detailed LC-MS in Table S3). MS files were imported into Skyline v23.1, an open-source software tool developed for MS quantitative analyses (https://skyline.ms/project/home/begin.view). A transition list was created for each glycan, consisting of possible B and Y type ions, as well as other known transitions identified by manual MS/MS assignment such as fragments produced by the Rapi-Fluor-MS tag. Targeted MS/MS filtering with a resolution of 60,000 was used to import transitions. The relative abundance (%) of each glycan was calculated by dividing the peak area of the sum of a glycan’s transitions by the total peak area of all quantified transitions for a given sample.

### One-way ANOVA

To identify potential significant glycan abundance changes, a one-way ANOVA (Analysis of Variance) was performed using webtool of Meta-boAnalyst (Version 6.0, https://www.metaboanalyst.ca/)^23^, which is a statistical test utilized to compare the means of multiple groups for significant differences. The relative abundance data of all the drug lots from LC-MS was groupedto six (Table 1, S1 and S2) in spreadsheet, which was usedas generic format of unpaired input and the module of StatisticalAnalysis [One Factor] was used. The input data were analyzed without filtering or normalization to preserve original variability. The one-way ANOVA was performed to evaluate differences in mean of the relative abundance levels across groups, with a significance threshold alpha factor of 0.05.

## Supplementary Material

SI

**Supplementary information** The online version contains supplementary material available at https://doi.org/10.1038/s44334-025-00058-5.

## Figures and Tables

**Fig. 1 |:**
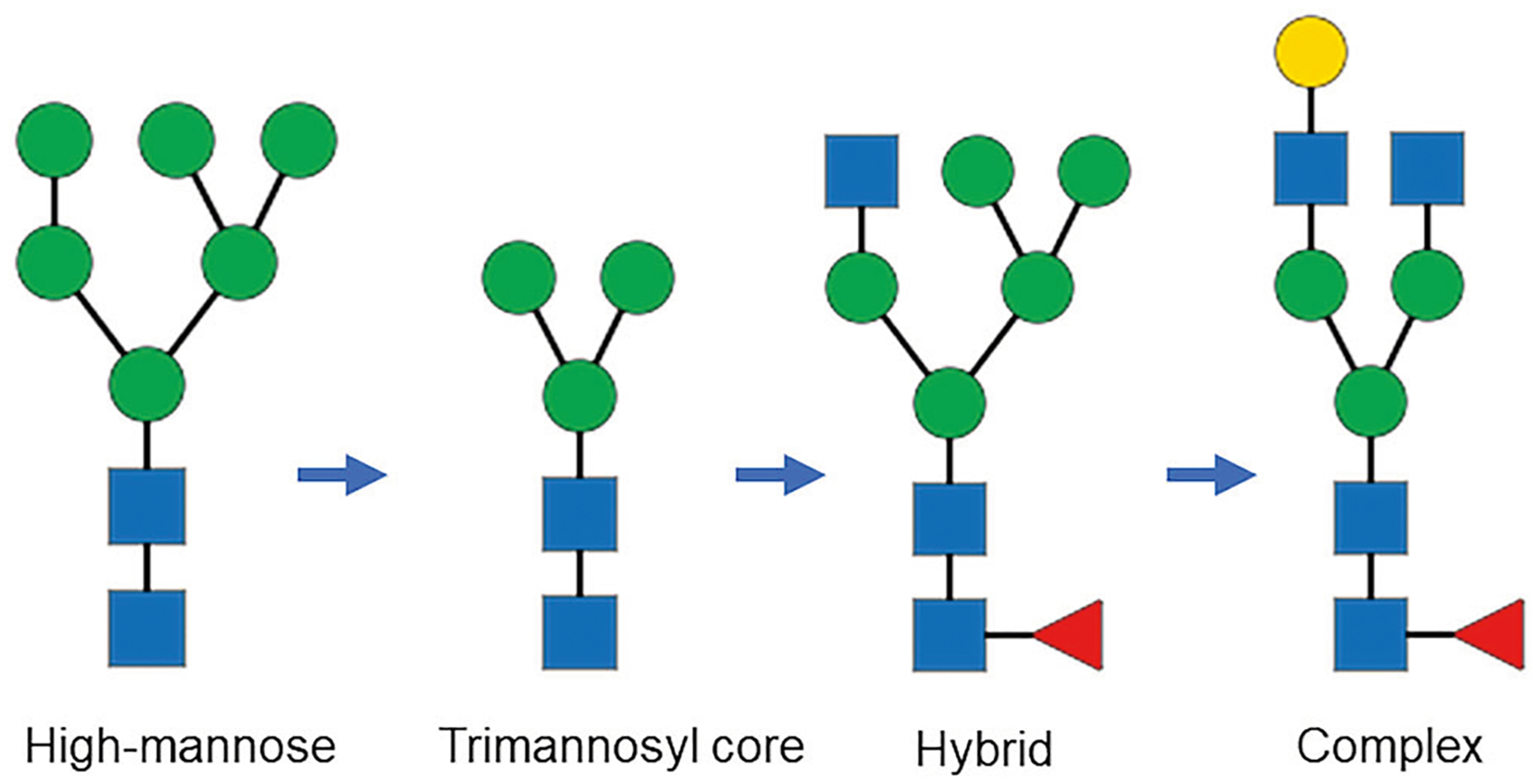
Representative glycan species illustrating the intracellular processing pathway. Glycans shown from left to right were high mannose M6,. trimannosyl core M3, hybrid FA1M5, and complex FA2[3]G1. Monosaccharides were represented by blue square for N-acetylglucosamine (GlcNac), green circle for mannose, yellow circle for galactose and red triangle for fucose.

**Fig. 2 |:**
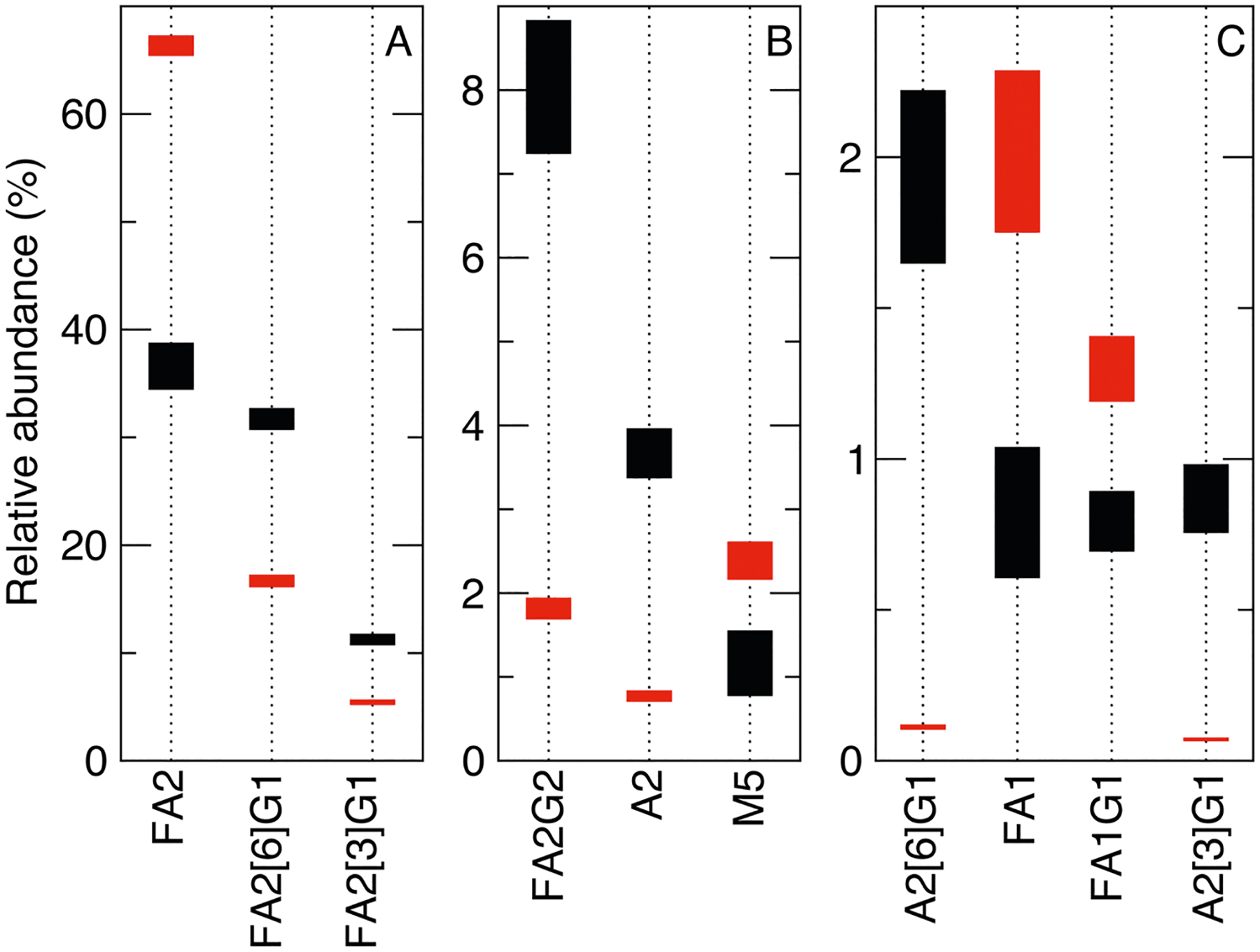
Range plots showing the relative abundances for the top glycans. Glycans of trastuzumab (black) and adalimumab (red) were ordered and grouped as (**A**) predominant glycans (>10%), (**B**) top major glycans (1–10%), and (**C**) remaining major and top minor glycans (0.1–1%) according to their relative abundances in trastuzumab (Table S4).

**Fig. 3 |:**
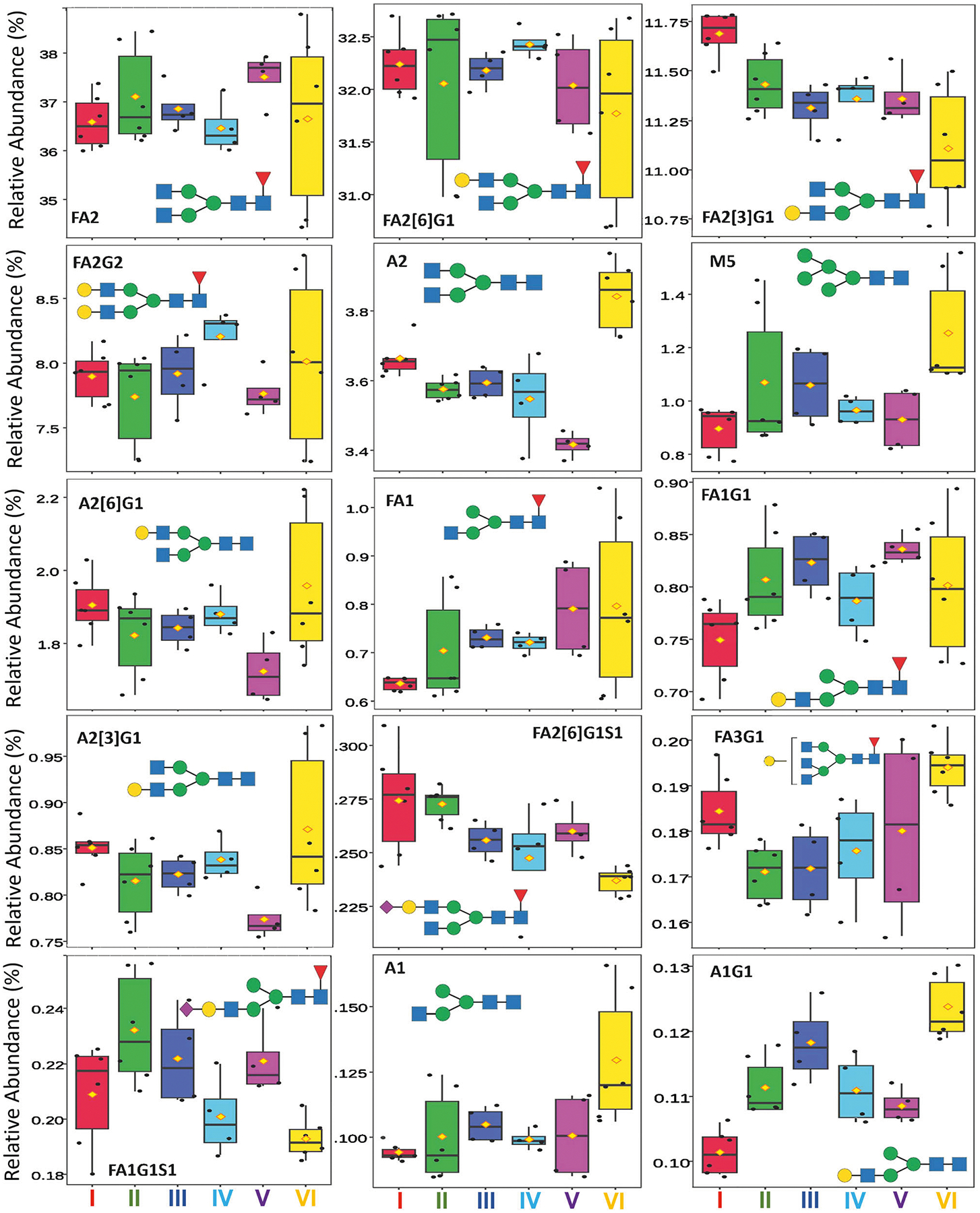
Box plots showing the relative abundance of top glycans in trastuzumab lot groups. The predominant (>10%), major (1–10%), and top minor (0.1–1%) glycans were shown following ANOVA analysis. Within each group, the line and diamond represent the median and mean values, respectively. The box height indicates the interquartile range (IQR), and the vertical whiskers represent the full data range excluding outliers.

**Fig. 4 |:**
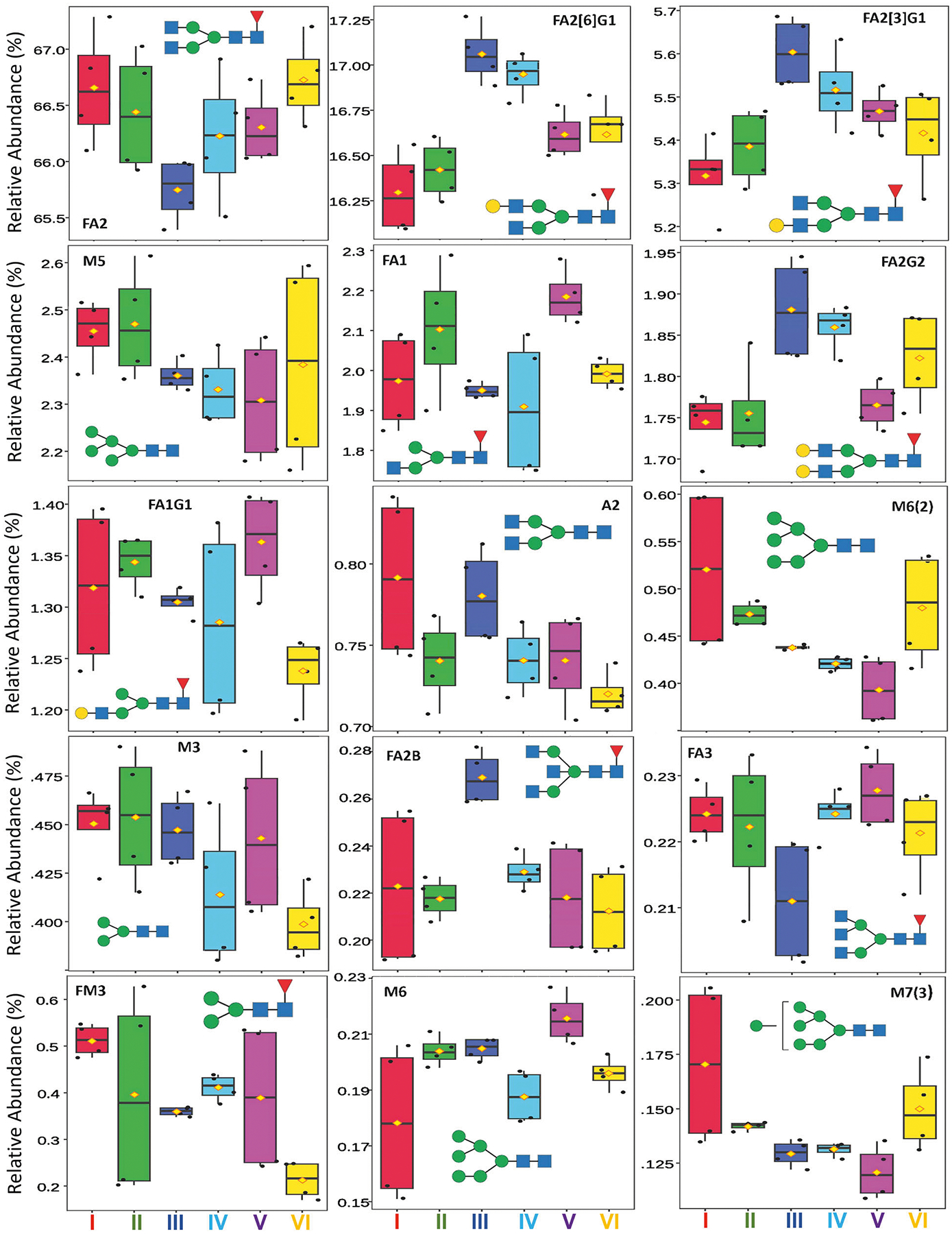
Box plots showing the relative abundance of top glycans in adalimumab lot groups. The predominant (>10%), major (1–10%), and top minor (0.1–1%) glycans were shown following ANOVA analysis. Within each group, the line and diamond represent the median and mean values, respectively. The box height indicates the interquartile range (IQR), and the vertical whiskers represent the full data range excluding outliers.

**Table 1 |:** Summary of mAb drug product lots grouped with expiry dates

	Trastuzumab		Adalimumab	
Groups	Number of lots	Date range of expiry	Number of lots	Date range of expiry
I	3	08/2020–02/2022	2	03/2019–07/2020
II	2	12/2022–02/2023	2	07/2020–04/2021
III	2	04/2023–08/2023	2	09/2021–09/2021
IV	2	09/2023–11/2023	2	03/2022–08/2022
V	2	12/2023–03/2024	2	10/2022–07/2023
VI	3	03/2024–09/2024	2	01/2024–06/2024

## Data Availability

All the data can be found in the Supplementary Information.
