# Supplementary material for: On the achievable consistency of glycan distribution in biomanufacturing of therapeutic mAbs
Source: NPJ Adv Manuf. Author manuscript; Available in PMC 2026 Feb 13. (PMC12895347; doi:10.1038/s44334-025-00058-5)
Supplement: SI [file NIHMS2145222-supplement-SI.pdf]

On the achievable consistency of glycan distribution in biomanufacturing of therapeutic mAbs

Hongbin Zhu 1, Joshua Shipman 1, Weiming Ouyang 2, Kang Chen 3*

1 Division of Pharmaceutical Quality and Research II, Office of Pharmaceutical Quality Research, Office of Pharmaceutical Quality, Center for Drug Evaluation and Research, U.S. Food and Drug Administration, St. Louis, MO, 63110, USA

2 Division of Pharmaceutical Quality and Research III, 3 Division of Pharmaceutical Quality and Research II, Office of Pharmaceutical Quality Research, Office of Pharmaceutical Quality, Center for Drug Evaluation and Research, U.S. Food and Drug Administration, Silver Spring, MD 20993, USA

*Corresponding author: kang.chen@fda.hhs.gov

Table S1: Trastuzumab drug lot list.

Table S2: Adalimumab drug lot list.

Table S3: LC/MS conditions.

Table S4: Trastuzumab results of range of change.

Table S5: Trastuzumab results of precision test.

Table S6: Adalimumab results of range of change.

Table S7: Adalimumab results of precision test.

Excel files:

SI-trastuzumab-data.xlsx,

SI-adalimumab-data.xlsx

Table S1. Group of the of Trastuzumab drug products in the six groups based on expiration dates.

Lot#_Prep#	Exp Date, YYYYMM	Group
B2008_1	202008	Group I
B2008_2	202008	Group I
3245566_1	202202	Group I
3245566_2	202202	Group I
3245565_1	202202	Group I
3245565_2	202202	Group I
3308198(vial#1)_1	202212	Group II
3308198(vial#1)_2	202212	Group II
3308198 (vial#2)_1	202212	Group II
3308198 (vial#2)_2	202212	Group II
3335977_1	202302	Group II
3335977_2	202302	Group II
3330042_1	202304	Group III
3330042_2	202304	Group III
3359911_1	202308	Group III
3359911_2	202308	Group III
3391472_1	202309	Group IV
3391472_2	202309	Group IV
3442253_1	202311	Group IV
3442253_2	202311	Group IV
3470109_1	202312	Group V
3470109_2	202312	Group V
3514960_1	202403	Group V
3514960_2	202403	Group V
3538470_1	202403	Group VI
3538470_2	202403	Group VI
3553969_1	202405	Group VI
3553969_2	202405	Group VI
3581428_1	202409	Group VI
3581428_2	202409	Group VI

Table S2. Group of the of Adalimumab drug products in the six groups based on expiration dates.

**Lot#_Prep#**	**Exp Date, YYYYMM**	**Group**
1080998_1	201903	Group I
1080998_2	201903	Group I
1108313_1	202007	Group I
1108313_2	202007	Group I
1110163_1	202007	Group II
1110163_2	202007	Group II
1118641_1	202104	Group II
1118641_2	202104	Group II
1123833_1	202109	Group III
1123833_2	202109	Group III
1124462_1	202109	Group III
1124462_2	202109	Group III
1133415_1	202203	Group IV
1133415_2	202203	Group IV
1140014_1	202208	Group IV
1140014_2	202208	Group IV
1151441_1	202210	Group V
1151441_2	202210	Group V
1157576_1	202307	Group V
1157576_2	202307	Group V
1163896_1	202401	Group VI
1163896_2	202401	Group VI
1179366_1	202406	Group VI
1179366_2	202406	Group VI

Table S3. LCMS parameters.

LC Conditions	LC Column	Waters Acquity BEH Amide column (130 Å, 1.7 µm, 2.1 × 150 mm)		
	Mobile Phases	A: 50 mM ammonium formate pH 4.4		
		B: Acetonitrile		
	Injection Volume	5 µl		
	Column Temp	45 °C		
	LC Gradient	Time (min)	B (%)	Flow Rate (µL/min)
		0	75	500
		30	71	500
		44	60	500
		45	0	250
		53	0	250
		53.5	75	500
		54.6	75	500
		60	75	500
General MS Conditions	Scan Type	PRM		
	Polarity	ESI+		
	Ion Source	HESI		
	Spray Voltage	4.2 KV		
	Ion Transfer Tube Temperature	300 C		
	Source Gas	40 for sheath gas, 10 for aux gas		
	Internal Mass Calibration	No		
	Full MS1	None		
	Targeted MS2 (PRM)	m/z 150–2000 at resolution of 60,000		
	NCE	35±10%		

Table S4 The range of change of each glycan within trastuzumab drug lots studied in Table S1.

**Glycan**	**Max**	**Min**	**Range**	**Population**	**P value**
FA2	38.798	34.431	4.367	Predominant	0.6712
FA2[6]G1	32.715	30.69	2.025		0.6127
FA2[3]G1	11.781	10.714	1.067		**0.001002**
FA2G2	8.834	7.239	1.595	Major	0.53842
A2	3.966	3.37	0.596		**1.12E-07**
M5	1.556	0.774	0.782		**0.027845**
A2[6]G1	2.222	1.647	0.575		0.11078
FA1	1.04	0.605	0.435		0.14703
FA1G1	0.894	0.693	0.201	Minor	0.079121
A2[3]G1	0.983	0.755	0.228		0.059435
FA2[6]G2S1	0.506	0.383	0.123		0.98323
A2G2	0.441	0.281	0.16		0.07891
FA2[3]G2S1	0.304	0.23	0.074		0.98926
FA2[6]G1S1	0.309	0.211	0.098		**0.003892**
FA3G1	0.203	0.157	0.046		**0.012442**
FA2G2S2	0.308	0.218	0.09		0.93818
FA1G1S1	0.256	0.18	0.076		**0.004317**
A1	0.166	0.085	0.081		**0.0111**
A1G1	0.13	0.098	0.032		**5.02E-07**
M6	0.129	0.056	0.073		0.09553
FA2[3]G1S1	0.17	0.122	0.048		**0.018077**
FA3	0.098	0.061	0.037	Trace	0.073286
M7	0.06	0.025	0.035		0.059657
M6A1	0.039	0.022	0.017		**0.000433**
FM3	0.048	0.02	0.028		0.072014
FA2G2Ga1	0.043	0.031	0.012		0.34261
M3	0.03	0.011	0.019		**0.000944**
M8(2)	0.029	0.011	0.018		0.084445
M8	0.034	0.022	0.012		**1.67E-06**
FA2[6]G2Sg1	0.034	0.026	0.008		0.43418
FA2G2S1Sg1	0.046	0.03	0.016		0.99323
FA2[6]G1Sg1	0.025	0.016	0.009		**0.003673**
FA2[3]G2Sg1	0.021	0.016	0.005		0.7777
M4	0.01	0.004	0.006		**0.049762**
FA2[3]G1Sg1	0.014	0.01	0.004		0.13638
A2[6]G2S1	0.016	0.01	0.006		**0.012393**
A2[3]G2S1	0.009	0.006	0.003		**0.006743**
A2[6]G1S1	0.009	0.006	0.003		0.20707
A2[3]G1S1	0.005	0.003	0.002		**0.001862**

Table S5 The relative abundance and experimental precision of trastuzumab based on the most recent lot 3581428.

Glycan	Relative Abundance (%)	Standard deviation (%)
FA2	39.026	0.318
FA2[6]G1	30.564	0.15
FA2[3]G1	10.426	0.167
FA2G2	7.275	0.064
A2	4.007	0.058
M5	1.749	0.026
A2[6]G1	1.602	0.016
FA1	1.057	0.013
FA1G1	0.859	0.014
A2[3]G1	0.738	0.01
FA2[6]G2S1	0.388	0.008
A2G2	0.276	0.003
FA2[3]G2S1	0.227	0.008
FA2[6]G1S1	0.213	0.004
FA3G1	0.195	0.003
FA2G2S2	0.185	0.013
FA1G1S1	0.181	0.005
A1	0.157	0.004
A1G1	0.144	0.001
M6	0.144	0.002
FA2[3]G1S1	0.121	0.003
FA3	0.086	0.001
M7	0.06	0.001
M6A1	0.046	0.001
FM3	0.045	0.001
FA2G2Ga1	0.03	0.001
M3	0.028	0.001
M8(2)	0.025	0.001
M8	0.024	0.001
FA2[6]G2Sg1	0.023	0.001
FA2G2S1Sg1	0.022	0.002
FA2[6]G1Sg1	0.016	0.001
FA2[3]G2Sg1	0.015	0.001
M4	0.01	0
FA2[3]G1Sg1	0.01	0
A2[6]G2S1	0.01	0
A2[3]G2S1	0.006	0
A2[6]G1S1	0.006	0
A2[3]G1S1	0.003	0

Table S6 The range of change of each glycan within adalimumab drug lots studied in Table S2.

**Glycan**	**Max**	**Min**	**Range**	**Population**	**P value**
FA2	67.288	65.393	1.895	Predominant	0.080781
FA2[6]G1	17.268	16.095	1.173		**6.14E-05**
FA2[3]G1	5.686	5.192	0.494	Major	**0.003727**
M5	2.614	2.16	0.454		0.39428
FA1	2.288	1.75	0.538		**0.029191**
FA2G2	1.945	1.685	0.26		**0.002452**
FA1G1	1.407	1.19	0.217		0.085217
A2	0.841	0.704	0.137	Minor	**0.032006**
M6(2)	0.597	0.361	0.236		**0.014082**
M3	0.49	0.38	0.11		0.089701
FA2B	0.282	0.192	0.09		**0.010383**
FA3	0.234	0.202	0.032		0.079434
FM3	0.628	0.17	0.458		**0.045155**
M6	0.227	0.151	0.076		**0.013157**
M7(3)	0.206	0.109	0.097		**0.017109**
A2[6]G1	0.12	0.103	0.017		**0.017998**
M7(2)	0.112	0.086	0.026		**0.038548**
M7	0.127	0.095	0.032		**0.021727**
FA3G1	0.118	0.102	0.016		0.074961
FM5A1	0.115	0.065	0.05		**0.016246**
FM5A1G1	0.091	0.063	0.028	Trace	**0.043449**
FA2BG1	0.116	0.076	0.04		**0.001919**
A2[3]G1	0.077	0.065	0.012		**0.027316**
M8	0.063	0.05	0.013		**0.013251**
FA1G1S1	0.073	0.037	0.036		**0.001808**
M4	0.066	0.047	0.019		**0.047651**
M8(2)	0.044	0.021	0.023		**0.028051**
FA2[3]G2S1	0.042	0.029	0.013		0.74589
A1	0.03	0.023	0.007		0.32599
A2G2	0.042	0.036	0.006		**5.48E-07**
A1G1	0.033	0.027	0.006		**0.004323**
M9	0.021	0.013	0.008		0.070764
FA2[6]G2S1	0.03	0.015	0.015		**0.0013**
M4(2)	0.025	0.017	0.008		0.33435
FA2[3]G1S1	0.025	0.018	0.007		0.43562
FA2[6]G1S1	0.023	0.009	0.014		**0.001304**
M5A1G1	0.016	0.012	0.004		0.13918
FM4	0.003	0.002	0.001		0.063442

Table S7 The relative abundance and experimental precision of adalimumab based on the most recent lot 1179366.

Glycan	Relative Abundance (%)	Standard deviation (%)
FA2	65.038	0.206
FA2[6]G1	16.827	0.135
FA2[3]G1	5.767	0.05
M5	2.593	0.08
FA1	2.208	0.024
FA2G2	1.9	0.018
FA1G1	1.289	0.005
A2	0.756	0.011
M6(2)	0.629	0.017
M3	0.423	0.005
FA2B	0.252	0.002
FA3	0.242	0.002
FM3	0.228	0.002
M6	0.226	0.004
M7(3)	0.198	0.01
A2[6]G1	0.134	0.002
M7(2)	0.124	0.002
M7	0.119	0.003
FA3G1	0.119	0.002
FM5A1	0.119	0.004
FM5A1G1	0.101	0.002
FA2BG1	0.095	0.001
A2[3]G1	0.083	0.001
M8	0.075	0.002
FA1G1S1	0.062	0.001
M4	0.06	0
M8(2)	0.042	0.002
FA2[3]G2S1	0.038	0.001
A1	0.038	0.003
A2G2	0.037	0
A1G1	0.03	0.001
M9	0.028	0.001
FA2[6]G2S1	0.028	0.001
M4(2)	0.026	0.002
FA2[3]G1S1	0.026	0
FA2[6]G1S1	0.021	0
M5A1G1	0.017	0.001
FM4	0.003	0
